# Enhancing the reliability of particulate matter sensing by multivariate Tobit model using weather and air quality data

**DOI:** 10.1038/s41598-023-40468-z

**Published:** 2023-08-12

**Authors:** Wan-Sik Won, Jinhong Noh, Rosy Oh, Woojoo Lee, Jong-Won Lee, Pei-Chen Su, Yong-Jin Yoon

**Affiliations:** 1 School of Mechanical and Aerospace Engineering, Nanyang Technological University, 50 Nanyang Avenue, Singapore, 639798 Singapore; 2 Department of Aerospace Industrial and Systems Engineering, Hanseo University, Taean, Chungcheongnam-do 32158 Republic of Korea; 3 Department of Mechanical Engineering, Korea Advanced Institute of Science and Technology (KAIST), 291 Daehak-ro, Yuseong-gu, Daejeon, 34141 Republic of Korea; 4 Department of Mathematics, Korea Military Academy, Seoul, 01805 Republic of Korea; 5 Department of Public Health Sciences, Graduate School of Public Health, Seoul National University, Seoul, 08826 Republic of Korea; 6 Observer Foundation, Seoul, 04050 Republic of Korea

**Keywords:** Environmental sciences, Engineering

## Abstract

Low-cost particulate matter (PM) sensors have been widely used following recent sensor-technology advancements; however, inherent limitations of low-cost monitors (LCMs), which operate based on light scattering without an air-conditioning function, still restrict their applicability. We propose a regional calibration of LCMs using a multivariate Tobit model with historical weather and air quality data to improve the accuracy of ambient air monitoring, which is highly dependent on meteorological conditions, local climate, and regional PM properties. Weather observations and PM_2.5_ (fine inhalable particles with diameters ≤ 2.5 μm) concentrations from two regions in Korea, Incheon and Jeju, and one in Singapore were used as training data to build a visibility-based calibration model. To validate the model, field measurements were conducted by an LCM in Jeju and Singapore, where R^2^ and the error after applying the model in Jeju improved (from 0.85 to 0.88) and reduced by 44% (from 8.4 to 4.7 μg m^−3^), respectively. The results demonstrated that regional calibration involving air temperature, relative humidity, and other local climate parameters can efficiently correct the bias of the sensor. Our findings suggest that the proposed post-processing using the Tobit model with regional weather and air quality data enhances the applicability of LCMs.

## Introduction

Rapid advances in computing systems and machine learning (ML) have increased the number of sensor technologies for real-time application by using abundant data and discovering hidden properties and patterns^1,2^. Data-driven approaches and scientific findings using big data analysis have shown great potential for applications in sensors and environmental industries^3–6^. Low-cost monitors (LCMs) are widely used in various types of urban areas to monitor air quality in real time. To improve the accuracy and applicability of LCMs, various environmental parameters have been proposed, showing promising improvements in sensing performance^7–9^.

Particulate matter (PM) is a key parameter of air quality and its measurement is required for accurate air quality monitoring. In particular, the concentration of fine particulate matter (PM_2.5_, fine inhalable particles with diameters ≤ 2.5 μm) is a reliable indicator of PM exposure, as it has a significant impact on mortality globally^10–12^. Regulatory authorities in charge of countries’ environmental policies have generally used reference instruments that operate based on gravimetry or beta ray attenuation. These reference measurement systems have very high accuracy and precision, but their installation and maintenance costs are high; thus, LCMs, and especially LCMs with improved sensors and incorporated Internet of Things (IoT) technology, are attractive alternatives for high spatial density PM monitoring^13–15^. Studies have shown that LCMs are reliable and exhibit high accuracy during laboratory testing as well as when calibrated in the field^16–19^. Conversely, other studies have concluded that LCMs are sensitive to climate parameters such as air temperature and relative humidity (RH); however, long-term measurements combined with post-processing or ML can correct the bias of the sensors^7,20^.

LCMs are advantageous because they are easy to install and can perform real-time high-resolution PM monitoring^21,22^; however, their performance varies among sensors, and rigorous scientific verification of their reliability has so far been insufficient for regulatory use^20,23,24^. Compared to reference measurement systems that operate based on a filter-based gravimetric method or beta-attenuation monitoring (BAM), LCMs have inherently low accuracy, as they estimate mass concentration indirectly based on optical measurements of light scattering; thus, uncertainties arise, which are associated with meteorological factors, background PM concentration, aerosol chemical composition, and aerosol size distribution, which compromise the reliability of the sensors^25,26^.

**Figure 1:**
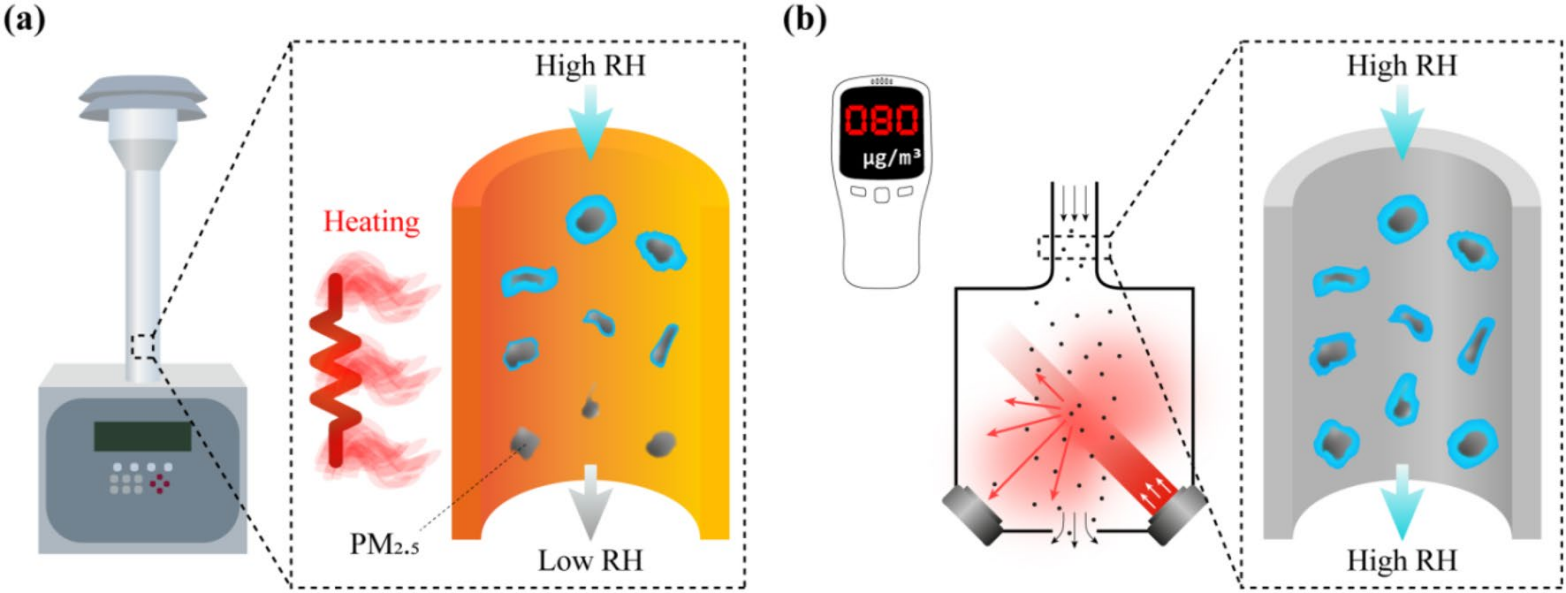
Schematic illustration of difference in PM_2.5_ measurement between (**a**) a BAM and (**b**) a LCM, specifically under highRH. The inlet tube in the BAM is heated to control the sampling conditions to RH 35%. Conversely, the LCM does not have air-conditioning function, resulting in increased light scattering through hygroscopic effect under high RH.

Another limitation is that LCMs typically do not perform conditioning of air temperature and RH of the sampled air. In the case of reference instruments, 24 h average air temperature and RH are conditioned during sampling between 20 and 23 °C and 30% and 40%, respectively^27–29^. LCMs typically do not control the sampling conditions (Fig. 1); hence, measurements are influenced by weather variations. The effects of air temperature and RH may be negligible in areas where PM concentrations are not significant, as well as during short-term field campaigns, depending on PM sources and background concentration^30,31^. However, weather may significantly impact the accuracy and repeatability of long-term monitoring in an area with highly variable PM concentrations during a high RH period due to PM_2.5_ hygroscopicity^26,32,33^. For these reasons, LCMs perform differently in different areas; for example, the same sensor has exhibited different accuracies in the USA compared to India or China^16,34,35^. Hence, regional calibration incorporating local climate information is an effective solution^36^.

As a part of the process of identifying regional differences that affect LCM performance, we present a post-processing method that uses airport visibility and PM_2.5_ concentration as training data sets. Visibility is an indicator of air quality that may include information on the concentration and hygroscopic growth of aerosol particles^37–39^; therefore, although airport visibility and PM concentration measured with an LCM are different parameters, they are both based on light scattering. Similar to an LCM estimating PM concentration from light scattering intensity, airport visibility has been reported based on light-source properties and transmission factors that contain information on light scattering around airport runways^40^. Airports are ideal for studying visibility because it is reported every hour and even more frequently under adverse conditions for aviation safety^41,42^. After collecting all weather data, it is possible to build a model to train the relationship between weather parameters and PM_2.5_, using a well-known basic light scattering principle^43^, which enables visibility prediction. Conversely, PM_2.5_ concentration can be estimated under various weather and visibility conditions by establishing empirical relationships regarding PM_2.5_, RH, and visibility^44–48^.

Here, we propose a regional calibration of LCMs that does not have an air-conditioning function using a multivariate Tobit model with airport weather and PM_2.5_ concentration data. We collected data from two middle-latitude regions in Korea, Incheon and Jeju, and one equatorial region in Singapore, thereby assembling a training dataset of visibility, weather parameters, and PM_2.5_ concentration. To calibrate the model to LCMs in different regions, we also conducted field measurements in Jeju and Singapore and compared the results before and after calibration, focusing on the differences in local climate that may affect LCM performance. Finally, we proposed better ways to use LCMs in different regions while overcoming their limitations during field measurements and could show that regional LCM calibration is feasible even without long-term field experiments.

## Results

### LCM dependency on RH

Figure 1 illustrates air conditioning in the case of BAM and LCM under high RH. Since the PM_2.5_ concentration, due to ambient moisture and hygroscopicity, may be uncertain, the BAM maintains dry conditions by evaporating water with a heater at the inlet^29,49^. PM_2.5_ concentration is precisely determined by beta-ray attenuation immediately after moisture has been removed from the sampled air. Conversely, the LCM detects light scattering by hygroscopic particles under high RH, which causes bias unless the LCM has undergone repeated calibration correctly.

**Figure 2:**
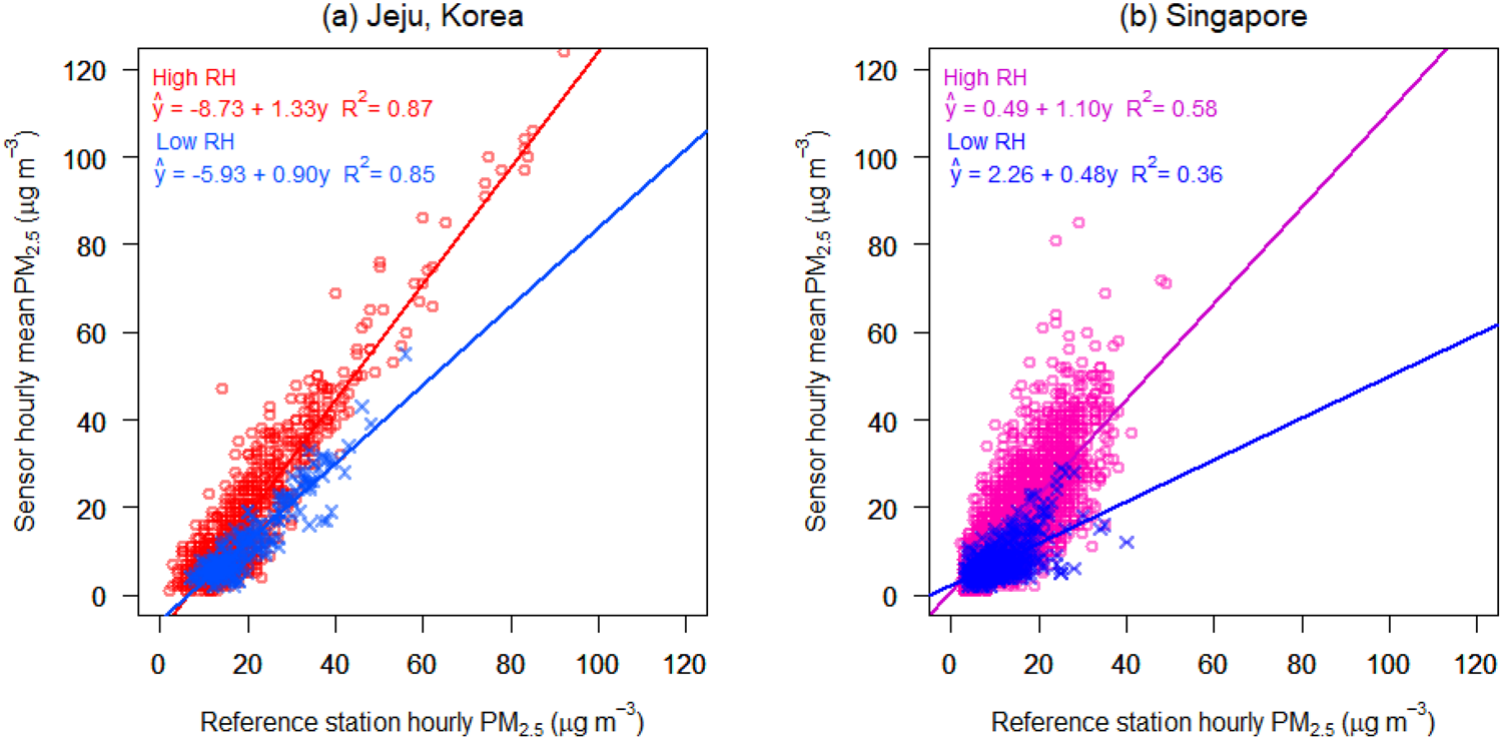
Different tendencies of the LCM caused by different RH in two different regions: Samples with high (> 70%) and low (< 40%) RH in (**a**) Jeju, Korea, and (**b**) Singapore. The LCM shows a positive bias of PM_2.5_ concentration in the case of high RH in both locations.

To validate the model, LCM field testing was conducted over seven months, from March 25 to October 26, 2019, in Jeju, and over 22 months, from December 1, 2020, to September 30, 2022, in Singapore (Figs. S4–S6 and Table S5). Figure 2 shows the PM_2.5_, concentrations by LCM, during high and low RH in the cases of Jeju and Singapore without post-processing calibration, criteria of high and low RH set as 70% and 40%, respectively. RH 70% and 40% are indicators of high and low RH, respectively. These criteria are not an absolute value but, rather, an arbitrarily set relative value. The vertical axis indicates the response of LCM at different RH conditions compared to the control conditions (RH 30‒40%) measured by the BAM, which is in a relatively low RH range. R^2^ indicates the coefficient of determination between the responses from LCM and BAM. The slope indicates the unit increase in the response from LCM to the unit increase in PM_2.5_ concentration from BAM.

During the LCM field testing, the average humidity in Jeju and Singapore was 65% and 69%, respectively. Field measurements in Jeju comprised of 2218 hourly observations over seven months. 9983 hourly observations were made in Singapore over a period of 22 months. Among the 2218 observations in Jeju, the number of data with RH above 70% and below 40% was 924 (red plots) and 233 (blue plots), respectively. Among 9983 observations in Singapore, the number of high and low RH data were 4556 (red) and 466 (blue), respectively.

While comparing high and low RH, the slope in the high RH case was greater than that in the low RH, both in Jeju and Singapore (1.33 and 1.10, respectively, for high RH). In terms of regional differences, measurements had a negative zero drift tendency in Jeju, whereas they had little zero drift tendency in Singapore. These results indicate that the sensor located in a more humid climate tends to yield a higher mass concentration, showing a bias between the reference station and the low-cost sensor under high RH. Therefore, the two different instruments in Fig. 2 represent the characteristics of the BAM and LCM in relation to Fig. 1, implying that the LCM without the inlet heater results in an increased bias under high RH conditions compared to the control RH (< 40%) of BAM.

### Airport and LCM in relation to light scattering

LCMs operate based on light scattering; accordingly, airport visibility is evaluated based on a light scattering sensor and optical observations of aerosols around the airport, hourly or at more frequent intervals^40^. RH and PM_2.5_ concentrations were the most correlated parameters with visibility (Fig. S3). The reported airport visibility and air quality data can elucidate the differences between the high and low RH cases during the LCM operation.

**Figure 3:**
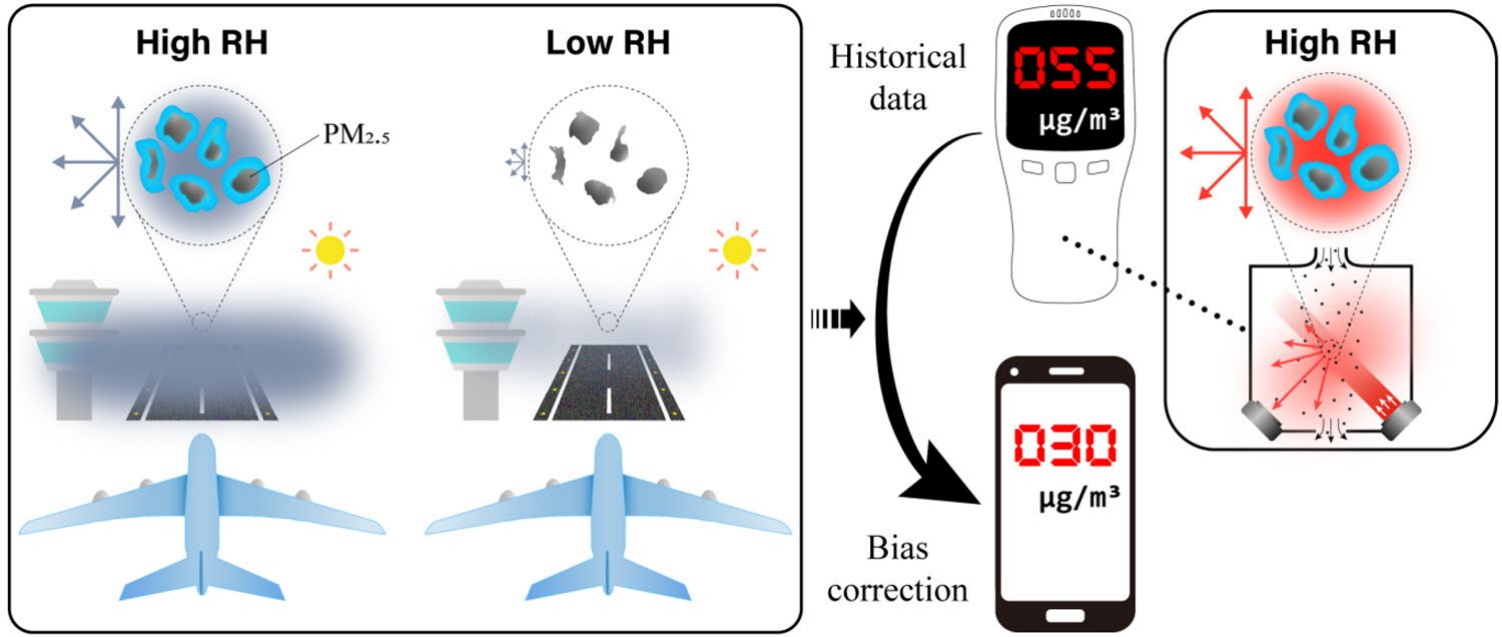
Illustrated flowchart of the calibration of PM_2.5_ concentration measurements by multivariate Tobit model using long-term visibility, weather observations, and air quality data from airports. The biased signals recorded by the LCMs can be corrected if the PM_2.5_ samples are conditioned to low RH (~ 30 to 40%); this is produced by the model using abundant historical data recorded at airports. Relationships between PM_2.5_ concentration, RH, and visibility are presented in Fig. S3.

Figure 3 shows an illustrated flowchart of the calibration by the multivariate Tobit model of the airport weather and PM_2.5_ concentration data to correct the LCM measurements under high RH, where the LCM sample scatters light from the transmitter, and the detector estimates the mass concentration from changing a signal such as voltage in response to the scattered light. Because hygroscopic particles scatter more light under humid conditions, light scattering and electrical signal reductions should be calculated if the RH is adjusted between 30 and 40% (the low-RH case was set to 35% in the selected model). Multi-annual airport weather observations were used for training and estimating the effect of RH on light scattering, and the result was applied to the calibration process.

A visibility prediction model was built using weather observations and PM_2.5_ concentration data from Incheon and Jeju in Korea and Singapore. The details of each model are presented in Tables S2 and S3, and the subsequent results are provided in Table S4. Won et al.^50^ showed that visibility prediction that considers PM_2.5_ concentration, meteorological parameters, and their relationships improved compared to other existing models. From the relationships between PM_2.5_ hygroscopicity, the visibility, and the extinction coefficient, airport observations and LCM measurements can be related to the hygroscopicity of PM_2.5_, which depends on RH. Reduced visibility under high RH indicates increased light scattering and absorption, which means an increased signal from aerosol particles under high RH, as illustrated in Fig. 3.

### PM effect on light scattering depending on regional climate

**Figure 4:**
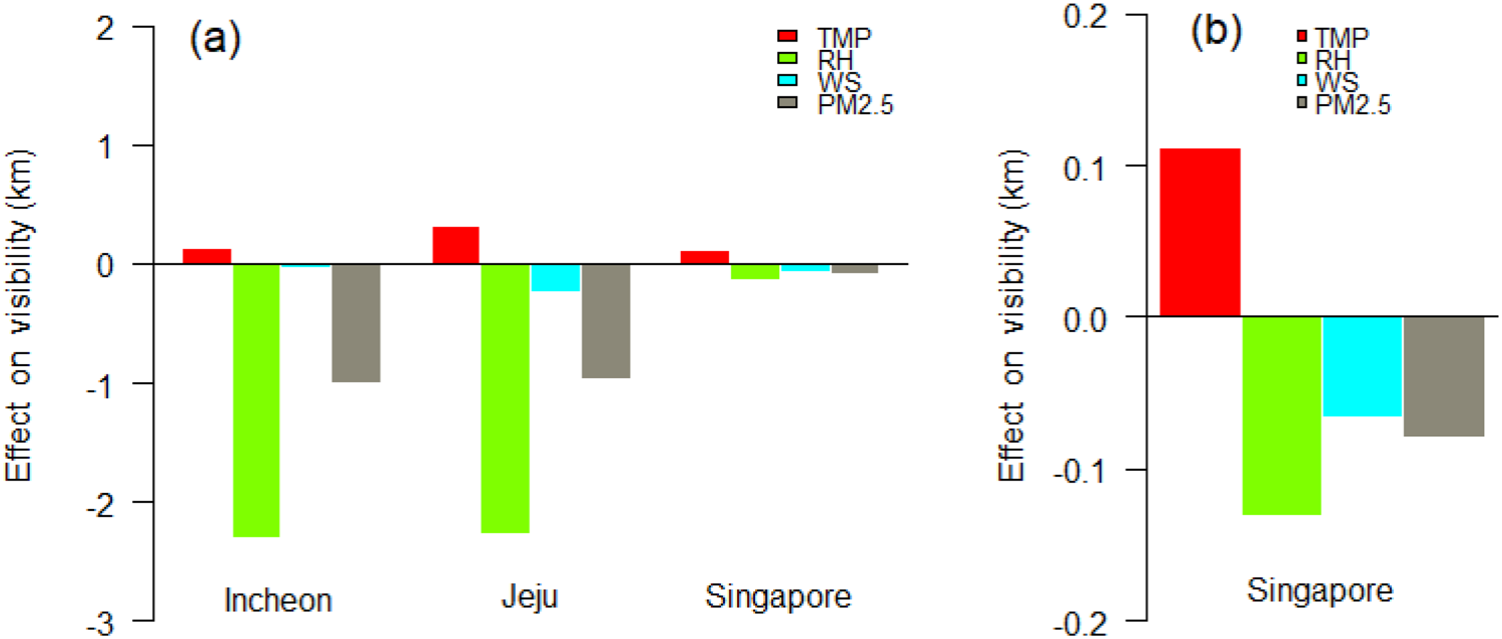
Regional visibility differences due to the effect of different weather parameters, i.e., air temperature (TMP), relative humidity (RH), and wind speed (WS), and PM_2.5_ concentration: (**a**) The effects in Incheon and Jeju are similar to each other, while those in Singapore are less pronounced due to the different regional climate. (**b**) The figure on the right shows an enlarged view in Singapore. The RH effect is negatively most pronounced, while the TMP effect is much more positively pronounced compared to the others in Singapore than in Incheon and Jeju.

Figure 4 shows the influence of air temperature (TMP), RH, and PM_2.5_ concentration on visibility in three different regions, which was determined by a multivariate Tobit model using airport and air quality data (the effects of all parameters on visibility estimated by the model are summarized in Table S4). In the Korean regions, RH has the most pronounced effect on visibility (− 2.31 and − 2.26 km in Incheon and Jeju, respectively), followed by PM_2.5_ concentration (− 1.00 and − 0.96 km in Incheon and Jeju, respectively), indicating that high RH and/or PM_2.5_ concentration is associated with decreased visibility, while TMP has a positive effect on visibility. Meanwhile, the influence of each parameter on visibility was smaller in Singapore (− 0.13 km for RH) than in the Korean regions (Fig. 4a). The difference between Singapore and Korea stems from the different local climates situated at the equator and middle latitudes, respectively. In Incheon and Jeju, the TMP variation was 53 °C (from − 16 to 37 °C) and 42 °C (from − 6 to 36 °C), while the RH variation was 90% (from 8 to 98%) and 88% (from 12 to 100%), respectively; while in Singapore, the TMP variation was 12 °C (from 22 to 34 °C) and the RH variation was 57% (from 43 to 100%) during the studied period (see Table S1). TMP and RH variations are not pronounced in the equatorial region; hence, their influence on visibility may be relatively small.

Figure 4b shows an enlarged view of the effect of weather parameters on visibility; the effects of RH, WS, and PM_2.5_ on visibility are − 0.13 km, − 0.06 km, and − 0.08 km in Singapore, respectively. Notably, unlike in Incheon and Jeju, the TMP effect on visibility is more pronounced in Singapore due to the local humid climate characteristics. The average RH during the study period was 81% in Singapore, and 62% and 67% in Incheon and Jeju, respectively (Table S1). At middle latitudes, RH variations were more pronounced than TMP variations. Conversely, at the equator, RH variation is quite small because the dew-point temperature is also relatively high, even when the TMP is high; thus, in Singapore, the influence of TMP on visibility is as pronounced as that of RH. The results from the model for the three regions reflect the similarities between neighboring mid-latitude regions and the climate characteristics at the equator.

### Calibration result depending on regional models

**Figure 5:**
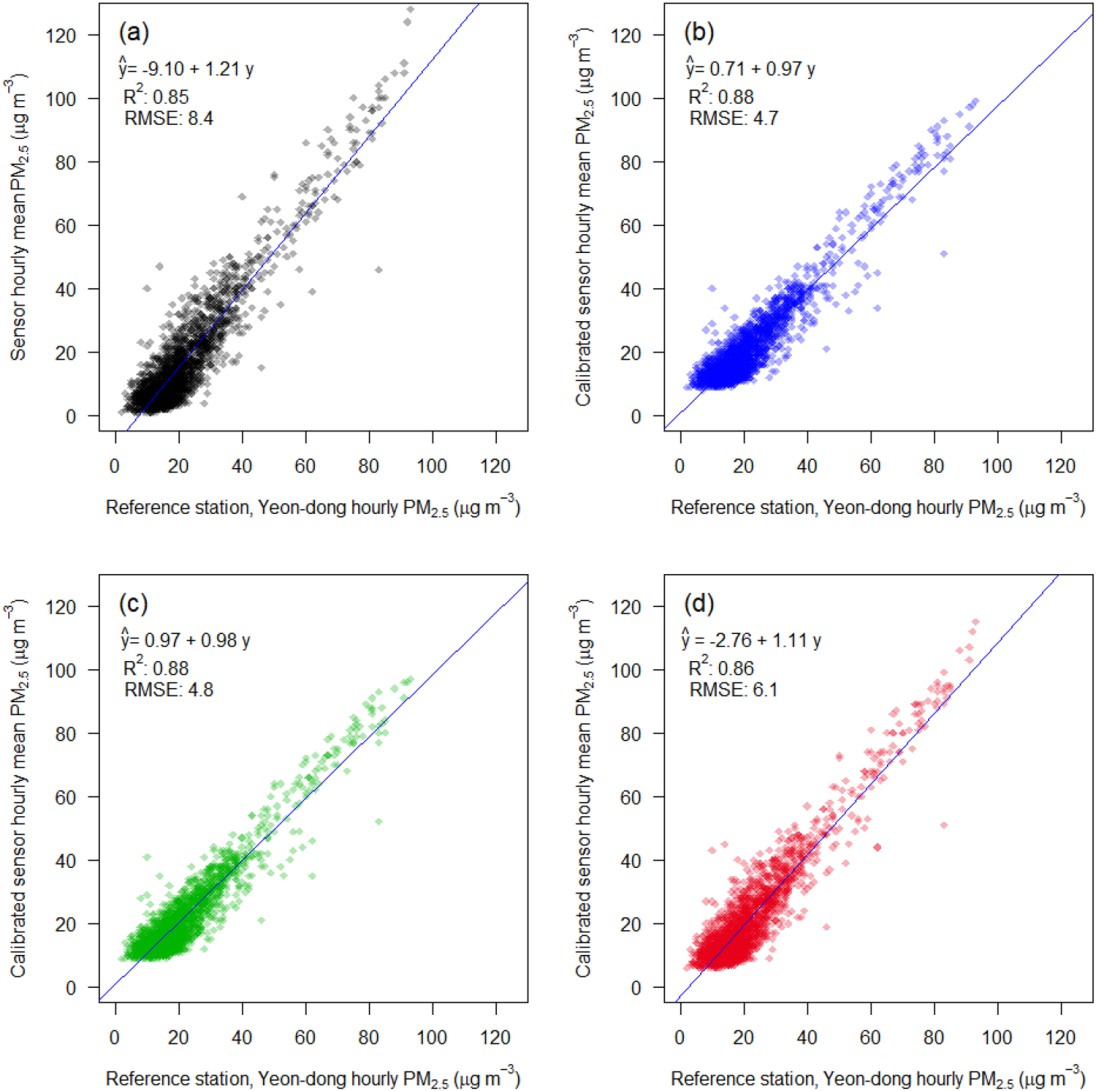
Scatterplots of hourly PM_2.5_ mass concentration of the LCM against the hourly measurements of the reference station in Jeju, Korea: (**a**) Raw PM_2.5_ data of the LCM; post-processed data according to the calibration method of the (**b**) Jeju, (**c**) Incheon, and (**d**) Singapore model.

LCM field testing combined with the calibration method was conducted in Jeju and Singapore (details are provided in “Methods” section and Fig. S5). Figure 5 shows the field measurement results for PM_2.5_, after applying the regional calibration by the model. Figure 5a presents the raw hourly PM_2.5_ concentration against the hourly measurements of the reference station, *Yeon-dong*, which is located near Jeju International Airport, and the other three panels present the calibrated PM_2.5_ concentration by the Jeju, Incheon, and Singapore models, respectively. Similar to the data in Fig. 2, raw LCM data exhibits mostly positive bias compared to those of the reference station since the PM_2.5_ concentration increases according to the 1.21 slope of linear regression. Conversely, low raw PM_2.5_ concentrations exhibit a negative bias compared to those of the reference station, indicating that the sensor has both zero and sensitivity drift in Jeju.

The three post-processed PM_2.5_ concentrations appear improved with linear regression slopes of 0.97, 0.98, and 1.11 respectively (Fig. 5b–d). Root Mean Square Error (RMSE) is 4.7, 4.8, and 6.1 after applying the Jeju, Incheon, and Singapore models; while the Jeju model exhibits, lowest error (Fig. 5b). The Incheon model result (Fig. 5c) is almost the same as that of the Jeju model. The Singapore model result (Fig. 5d) also shows improved accuracy; nevertheless, it exhibits a tendency toward higher values compared with the Jeju and Incheon model results.

PM_2.5_ measurements using LCM in Singapore and the calibrated results from the three regional models are shown in Fig. S8. Similar to Fig. 5, the plots from Jeju and Incheon are similar. The Singapore model result is different from the Jeju and Incheon model results, with a positive bias still remaining. It can be seen from the empirical relation stemming from the middle latitudes^46^, the extinction coefficient at 80–90% RH and average PM_2.5_ concentration of 22 μg m^−3^ is about 10–18 M m^−1^ (see “Methods” section, Eq. 3), while a study on hygroscopicity and visibility reported a value of 5.7–7.0 M m^−1^ in Singapore^51^. This difference may be reflected in the model, resulting in a more pronounced change after the calibration in Jeju than in Singapore.

All field measurement results over seven months in Jeju and 22 months in Singapore are summarized in Table 1. The average PM_2.5_ concentration over the measurement period in Singapore (13.0 μg m^−3^) is considerably less than that in Jeju (21.2 μg m^−3^). The mean PM_2.5_ concentration exhibits negative bias in Jeju and Singapore with 16.6 μg m^−3^ and 12.3 μg m^−3^, respectively. Both field experiments in Jeju and Singapore show that the mean PM_2.5_ concentration after calibration by the model (20.8–21.6 μg m^−3^ and 13.1–13.3 μg m^−3^, respectively) is close to that of the reference station (21.2 μg m^−3^ and 13.0 μg m^−3^ respectively). The R^2^ between the LCM and reference-station measurements is smaller in Singapore (0.51) than in Jeju (0.85), which may be due to the relatively low average concentration and relatively long distance between the reference station and the LCM in Singapore (Fig. S5). Regarding the results after calibration by the Jeju and Incheon models, RMSEs are nearly similar (4.7–4.8 μg m^−3^ in Jeju and 4.4 μg m^−3^ in Singapore) while the normalized error is smaller in Jeju (17%) than in Singapore (26%). The error of Jeju model is the lowest for field testing of Jeju and Singapore (4.7 μg m^−3^ and 17%; 4.4 μg m^−3^ and 26% respectively). However, the Jeju model has the lowest linear regression slope of 0.53 for field testing of Singapore, which means that the greater the ambient PM_2.5_ concentration, the greater the RMSE by the model. For example, in the case of high PM_2.5_ concentrations (in excess of 30 μg m^−3^ ), RMSE of the Singapore model is the lowest (9.7 μg m^−3^) while that of the Jeju model (12.3 μg m^−3^) is still as high as raw data (12.5 μg m^−3^).

**Table 1:** Mean PM_2.5_ concentration, coefficient of determination (R^2^), slope, RMSE, and normalized error between PM_2.5_ concentration measured by the LCM and measurements of the reference station over seven months (March to October 2019) in Jeju and 22 months (December 2020 to September 2022) in Singapore: each raw LCM value is compared with the respective calibrated values produced by three calibration methods developed by multivariate Tobit model using historical weather and air-quality data from Jeju, Incheon, and Singapore, respectively.

	Reference Station	Raw data	After calibration by each model		
			Jeju	Incheon	Singapore
Jeju					
Mean (μg m^−3^)	21.2	16.6	21.2	21.6	20.8
R^2^		0.85	0.88	0.88	0.86
Slope		1.21	0.97	0.98	1.11
RMSE (μg m^-3^)		8.4	**4.7**	4.8	6.1
Normalized error (%)		33	**17**	**17**	22
Singapore					
Mean (μg m^−3^)	13.0	12.3	13.1	13.5	13.3
R^2^		0.51	0.51	0.53	0.50
Slope		0.91	0.53	0.56	0.84
RMSE (μg m^−3^)		5.8 (12.5*)	**4.4** (12.3*)	**4.4** (11.7*)	5.5 (9.7*)
Normalized error (%)		33	**26**	**26**	31

Bold font indicates lowest errors.

*In case of high PM_2.5_ concentrations (in excess of 30 μg m^−3^ from the reference station in Singapore).

**Figure 6:**
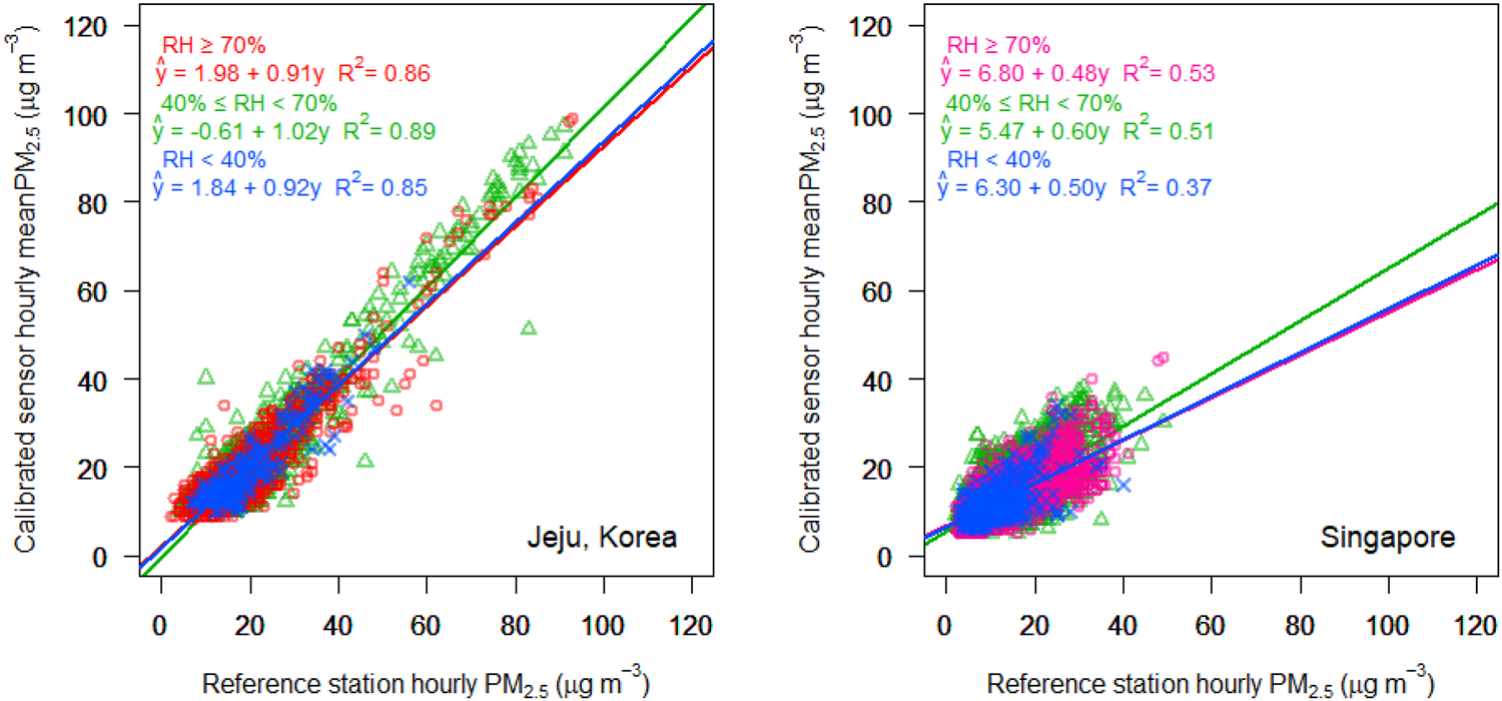
RH and TMP-adjusted PM_2.5_ concentrations by the calibration model, namely Jeju model, against those of the reference station for the different RH ranges in Jeju and Singapore. Data under different RH levels (low, moderate, and high RH) are shown in blue, green, and red, respectively.

Plots of the calibrated PM_2.5_ mass concentrations for different RH levels, are shown in Fig. 6. The RH and TMP-adjusted results for high RH exhibit a reduced sensor bias compared to the raw data shown in Fig. 2; the linear regression slopes of 0.91–1.02 in Jeju show that the calibration method can efficiently correct the bias of the sensor, regardless of the RH levels.

### Implications of modeling for regional calibration

The present study proposes a regional LCM calibration method that estimates PM_2.5_, which is typically sensitive to weather, using a multivariate Tobit model with airport weather and air quality data. Instead of air conditioning to 20–23 °C TMP and 30–40% RH, this method predicts PM_2.5_ concentration assuming that the LCM regulates TMP and RH at 21.5 ℃ and 35%, respectively. The validity of this airport weather-based calibration method was verified by constructing models for three regions, namely Incheon and Jeju in Korea at middle latitudes and Singapore at the equator, and complementary field measurements were conducted for several months in Jeju and Singapore.

The main argument against LCMs is that their accuracy is still not sufficiently high and is sensitive to the background concentration and regional environment, as also reported in the present study^16,26,32,34,35^. Although several studies have elaborated on the PM_2.5_ hygroscopic properties and their influence on LCM performance^26,32,33^, other studies have reported reduced TMP and RH effects on LCM performance during field calibration^30,52^, which may be due to relatively short measurement periods, low average concentrations, and less pronounced TMP and RH variations in these regions. This study presents a novel approach for understanding regional differences by performing regional calibration using visibility-prediction models and LCM field testing in two different regions.

Considering that visibility is a simple indicator of air quality, Molnár et al.^39^ showed that visibility-based PM hygroscopic growth is in good agreement with filter-based mass growth rate and can be applied to low-cost PM monitoring. Datta et al.^19^ showed that a calibration equation can be applied to another LCM in the network of the region because TMP, RH, and PM_2.5_ concentration trends in the region are similar, which is in line with the findings of this study, which showed that the Jeju model is most effective in field testing in Jeju. Zusman et al.^36^ showed that regional calibration may increase LCM reliability because meteorological conditions and PM sources may differ from one region to another. Onal et al.^3^ showed that the IoT Big Data framework and machine learning can identify regional climate differences from complex datasets. The regional calibration method using the Tobit model with multi-annual visibility, meteorological factors, and air quality data in this study can be supported by these studies because it reflects various local factors affecting LCM performance.

Routinely correcting the bias of LCMs using filter-based mass concentrations as a reference is an easy way to increase accuracy, but this is not always applicable, as in the case of variable PM concentrations/compositions and meteorological conditions. Such a correction is also not practically feasible because of the difficult acquisition of long-term observations in all regions.

As an attempt to focus on LCM air conditioning, similar to reference instruments, and regional calibration using a multivariate Tobit model, the significance of this study is as follows. First, we showed that there is abundant data for post-processing and generating a calibration model. The multivariate model generally requires as much data as possible; and many airports and air quality authorities possess decades of weather and PM_2.5_ concentration data. We developed a regional calibration formula by comparing several years of airport visibility reported every hour with PM sensor measurements, showing how post-processing techniques can be applied to airport meteorological data and air quality measurements around the world. Second, we demonstrated that the calibration method can reflect local characteristics without requiring long-term field testing. The proposed method can reproduce an effect similar to that of creating empirical relations through long-term field experiments by building a visibility-prediction model using accumulated historical data. Third, the accuracy of PM monitoring, which is region-dependent, was quantified using a specific type of LCM instrument. By comparing two regions with different climate characteristics, located in the middle latitudes and equator respectively, we revealed the LCM requirements for calibration according to the local climate parameters for more reliable ambient air monitoring. LCMs still have limitations in terms of accuracy and reliability, but their potential is invaluable, when combined with advances in postprocessing methodologies^7,8,20^.

East Asia has been experiencing increased PM_2.5_ concentrations due to climate change and related stagnant atmospheric conditions^53^. Southeast Asia, in particular, currently experiences severe haze every few years and faces the major challenge of mitigating any damage from such climate crises^54,55^. Future investigations should focus on these high-impact pollution events, and the use of LCMs for this purpose should allow more communities to have easy access to air quality information. The proposed regional calibration of the postprocessing method can enhance the applicability of outdoor air quality monitoring using LCMs.

## Methods

### Data

To establish relationships between meteorological parameters and PM_2.5_ concentration, airport weather observations and PM_2.5_ concentration data were collected from two regions in Korea, Incheon and Jeju, and one in Singapore (Figs. S1 and S2)^56,57^. The data cover four years, from January 2015 to December 2018 at Incheon and Jeju airports, and two years and six months, from April 2020 to September 2022 at Changi Airport in Singapore. Airport observations consist of hourly wind direction and wind speed (WS), visibility, *‘present weather’* (WX)^40^, air temperature (TMP), and dew-point temperature (DPT)^58^. Relative humidity (RH) was calculated using the TMP–DPT relation equation^59^. The selected air-quality monitoring stations are situated five kilometers northeast of Incheon airport, three kilometers south of Jeju airport, and five kilometers west of Changi airport in Singapore respectively; in all cases, the PM_2.5_ concentration was regularly monitored by reference instruments. The ranges of the meteorological parameters and PM_2.5_ concentration are summarized in Table S1. The correlation coefficients between the variables and their scatter plots are shown in Fig. S3.

### Modeling

The TMP, RH, WS, WX, and PM_2.5_ concentration are used as explanatory variables to predict visibility, which is determined as follows:

1 $$ Z_{{x_{i} }} = \frac{{x_{i} - \overline{{x_{i} }} }}{{s_{{x_{i} }} }} $$

where ${Z}_{{x}_{i}}$ is the standardized explanatory variable of ${x}_{i}$, ${x}_{i}$ represents the explanatory variable (i.e., meteorological parameters or PM concentration) for observation $i$, $\overline{{x}_{i}}$ is the mean value of the explanatory variable, and ${s}_{{x}_{i}}$ is the standard deviation of ${x}_{i}$. Since visibility observations in airports have an upper limit of 9999 m, it has right-censored data characteristics; therefore, the Tobit model is utilized as an post-processing tool with vector generalized linear model (VGLM) function in the programming language R^60,61^. The Tobit model is expressed as follows:

2 $$ y_{i} = \left\{ {\begin{array}{*{20}l} {Z_{{x_{i} }} \beta + \varepsilon_{i} } \hfill \\ {u } \hfill \\ \end{array} } \right. \begin{array}{*{20}c} {(y_{i} < u)} \\ {(y_{i} \ge u)} \\ \end{array} $$

where ${y}_{i}$ is the airport visibility for observation $i$, ${Z}_{{x}_{i}}$ represents the predictor variables for observation $i$, $\beta$ is a vector of regression parameters, ${\varepsilon }_{i} \sim N(0,{\sigma }^{2})$ is a random error for observation $i$, and $u$ is the censoring value, that is 9999 m. The selected parameters that constitute the model and subsequent results are summarized in Tables S2, S3, and S4. The RH and PM_2.5_ concentration have the greatest influence on the extinction coefficient; TMP and WS also has a significant effect on visibility^62,63^. The relationships between PM_2.5_ concentration, and weather parameters have also been incorporated into the model^50^.

### Calibration equations

**Figure 7:**
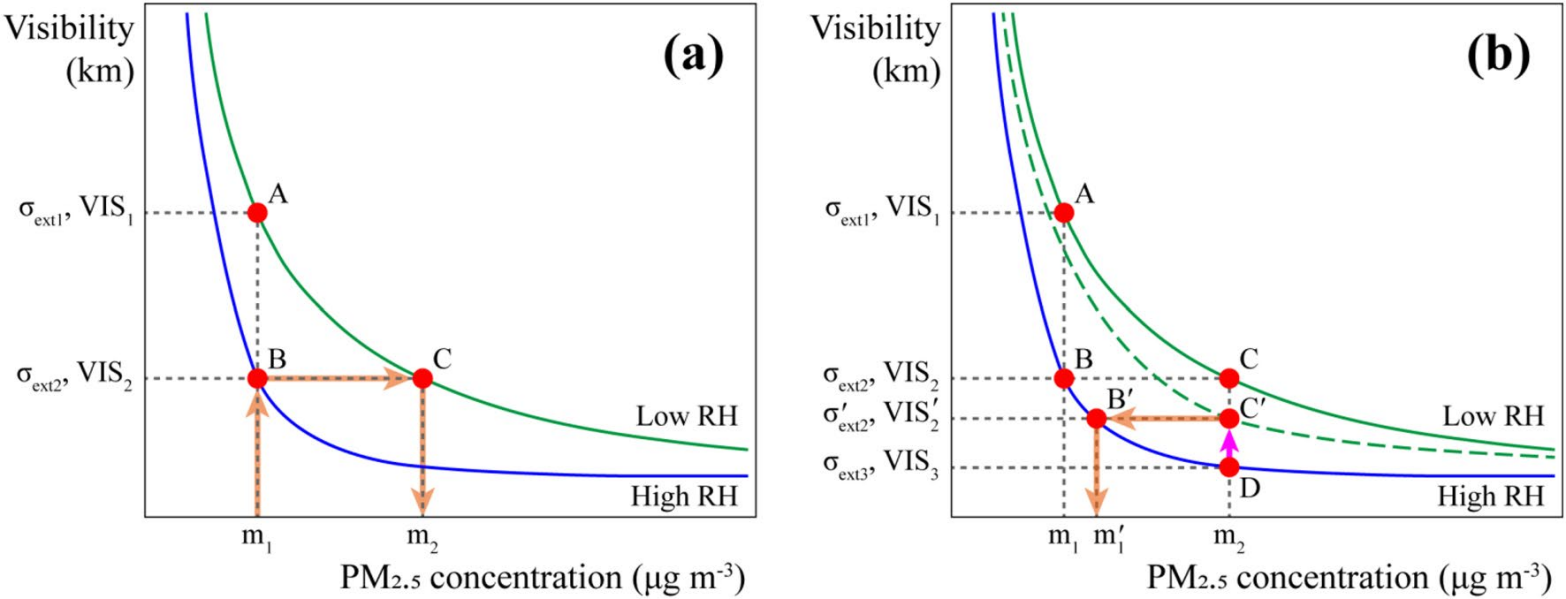
Schematic diagram of the proposed calibration method using visibility prediction by relative humidity (RH) air conditioning: (**a**) The graph shows the process of misreading m_1_ as m_2_ under high RH. (**b**) The arrows indicate how to correct the misread PM_2.5_ concentration on the equivalent curve of visibility, PM_2.5_ concentration, and RH, by predicting the change in visibility while assuming sample air conditioning in a LCM.

The predicted visibility from the above-mentioned model corresponds to the extinction coefficient in the equation below, which is derived from field measurements in various PM concentration ranges^46^:

3 $$ \sigma_{{ext_{i} }} \left( {RH_{i} } \right) = 3.97 \times PM_{{2.5_{i} }} \times \left( {1 + 8.8 \times \left( {\frac{{RH_{i} }}{100}} \right)^{9.7} } \right) + 0.62 \times PM_{{2.5_{i} }} + 25 $$

where ${{\sigma }_{ext}}_{i}$ is the extinction coefficient for observation $i$; and the visibility corresponding to ${{\sigma }_{ext}}_{i}$ is expressed as follows^43^:

4 $$ VIS_{i} = \frac{3.912}{{\sigma_{{ext_{i} }} }} $$

where ${VIS}_{i}$ is visibility (km) for observation $i$. Subsequently, we can determine visibility from Eqs. (3) and (4) using RH and PM_2.5_ concentration. For example, VIS_2_ in Fig. 7 represents visibility at a PM_2.5_ concentration of m_2_ under low RH. If the LCM yields m_2_ μg m^-3^ under high RH, we find the expected visibility variation (i.e., $\overline{DC{\prime}}$ in Fig. 7) by assuming that TMP and RH change to 21.5 °C and 35%, respectively, to calibrate the misread concentration. The expected visibility variation due to changes in TMP and RH was calculated using the model as follows:

5 $$ \Delta VIS_{{TMP_{i} }} = \left( {Z_{{21.5\,^\circ {\text{C}}}} - Z_{{TMP_{i} }} } \right) \times \left( {\beta_{TMP} + \beta_{{TMP:PM_{2.5} }} \times Z_{{PM_{{2.5_{i} }} }} } \right) $$

6 $$ \Delta VIS_{{RH_{i} }} = \left( {Z_{35\% } - Z_{{RH_{i} }} } \right) \times \left( {\beta_{RH} + \beta_{{RH:PM_{2.5} }} \times Z_{{PM_{{2.5_{i} }} }} } \right) $$

7 $$ VIS_{i}^{\prime } = VIS_{i} + \Delta VIS_{{TMP_{i} }} + \Delta VIS_{{RH_{i} }} $$

where ${{VIS}_{i}}{\prime}$ is the visibility predicted by the change in TMP and RH to 21.5 °C and 35%, respectively. Subsequently, a new extinction coefficient, ${{\sigma }_{ext}}_{i}{\prime}$, was obtained using Eq. (4). Finally, the calibrated concentration, ${{{PM}_{2.5}}_{i}}{\prime}$, was calculated using Eq. (3). It is the calibrated concentration ${\text{m}}_{1}^{\prime }$ (Fig. 7b) by air-conditioning TMP and RH to 21.5 °C and 35%, respectively. According to these equations, increased visibility changes due to air conditioning lead to more pronounced changes in the extinction coefficient, eventually resulting in high PM_2.5_ concentration in need of calibration. The empirical formula consists of RH and PM_2.5_ concentration, but the model enhances prediction performance by considering the effects of TMP and RH as well.

Figure 7 shows the process of correcting a misread PM_2.5_ concentration using visibility prediction under high RH. The two curves depict empirical relationships between RH, PM_2.5_, and visibility under high and low RH. The orange arrows in Fig. 7a indicates the sequence by which the LCM misreads the actual PM_2.5_ concentration, m_1_, as m_2_. Low PM_2.5_ concentration of m_1_ corresponds to high visibility of VIS_1_ under low RH (point A). Under high RH, the m_1_ concentration has a higher extinction coefficient, ${\sigma }_{{ext}_{2}}$, resulting in lower visibility of VIS_2_, which results in the LCM calibrated according to low RH by the manufacturer indicating a higher PM_2.5_ concentration of m_2_ (point C). Conversely, when the LCM indicates a PM_2.5_ concentration of m_2_ under high RH conditions, it should be calibrated to m_1_; hence, an LCM bias by RH can be corrected using the empirical relations if it is already known in the monitoring area; however, it is unknown in most cases.

Figure 7b shows the calibration process. From the empirical relation, the difference in visibility between high and low RH at the PM_2.5_ concentration of m_2_ is $\overline{DC}$, and the difference in PM_2.5_ concentration between high and low RH at VIS_2_ is $\overline{BC}$. However, because the empirical relation varies depending on regional and environmental conditions, it is impractical to construct every empirical relation in all regions where LCMs operate. Here, we calculate the anticipated visibility difference ($\overline{DC{\prime}}$) between high and low RH at PM_2.5_ concentration of m_2_ using the model. Subsequently, the difference in PM_2.5_ concentration before and after calibration is $\overline{B{\prime}C{\prime}}$, which is dependent on $\overline{DC{\prime}}$. For example, in the Jeju case on March 29, 2019, 06:00 KST (i.e., Korea standard time), $\overline{DC}$ from Fig. 7b was calculated as 4.0 km according to the empirical relation, and accordingly the PM_2.5_ concentration was calibrated as 52 μg m^−3^ (m_1_). Conversely, $\overline{DC{\prime}}$ is calculated as 3.0 km by the model, assuming that the sampled air in the LCM is conditioned at 21.5 °C TMP and 35% RH, thereby producing a final calibrated value of 57 μg m^−3^ (${\text{m}}_{1}^{\prime }$). (Table S6).

As described above, the proposed calibration method can be applied in a region where the empirical relation is unknown, but has sufficient historical weather and air quality data for predicting visibility.

### Supplementary Information

Supplementary Information.

## Data Availability

The datasets used and/or analysed during the current study available from the corresponding author on reasonable request.
