# Supplementary material for: Enhancing the reliability of particulate matter sensing by multivariate Tobit model using weather and air quality data
Source: Sci Rep. 2023 Aug 12;13:13150. doi: 10.1038/s41598-023-40468-z (PMC10423292; doi:10.1038/s41598-023-40468-z)
Supplement: Supplementary file 1 — Supplementary Information. [file 41598_2023_40468_MOESM1_ESM.docx]

**Supplementary Material**

Enhancing the reliability of particulate matter sensing by multivariate Tobit model using weather and air quality data

Wan-Sik Won^1,2^, Jinhong Noh^3^, Rosy Oh^4^, Woojoo Lee^5^, Jong-Won Lee^6^, Pei-Chen Su^1*^, Yong-Jin Yoon^1,3*^

^1^ School of Mechanical and Aerospace Engineering, Nanyang Technological University, Singapore

^2^ Department of Aerospace Industrial and Systems Engineering, Hanseo University, Chungcheongnam-do 32158, Republic of Korea

^3^ Department of Mechanical Engineering, Korea Advanced Institute of Science and Technology (KAIST), Daejeon 34141, Republic of Korea

^4^ Department of Mathematics, Korea Military Academy, Seoul 01805, Republic of Korea

^5^ Department of Public Health Sciences, Graduate School of Public Health, Seoul National University, Seoul 08826, Republic of Korea

^6^ Observer Foundation, Seoul 04050, Republic of Korea

^*^Corresponding authors

Yong-Jin Yoon – Email: yongjiny@kaist.ac.kr

Pei-Chen Su – Email: peichensu@ntu.edu.sg

Part I. Modeling (Visibility prediction)


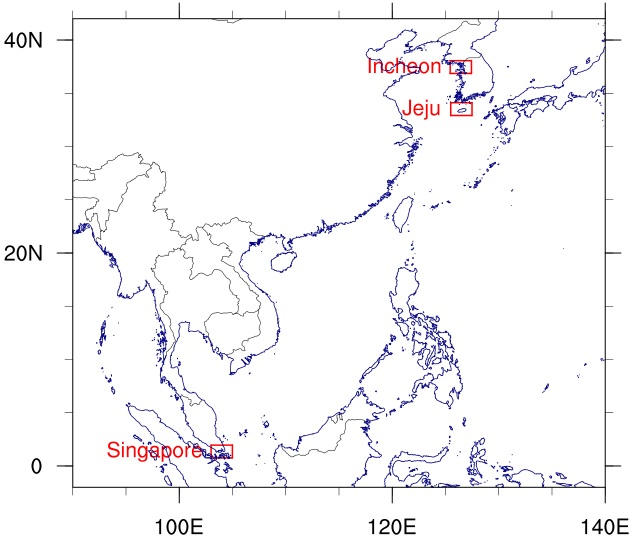


**Figure S1.** The three different regions—Jeju and Incheon in Korea and Singapore—included in the study.


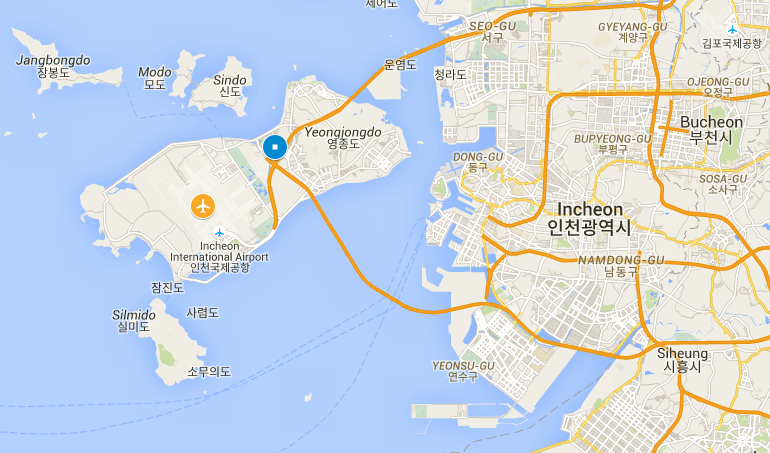


(a) Incheon, Korea


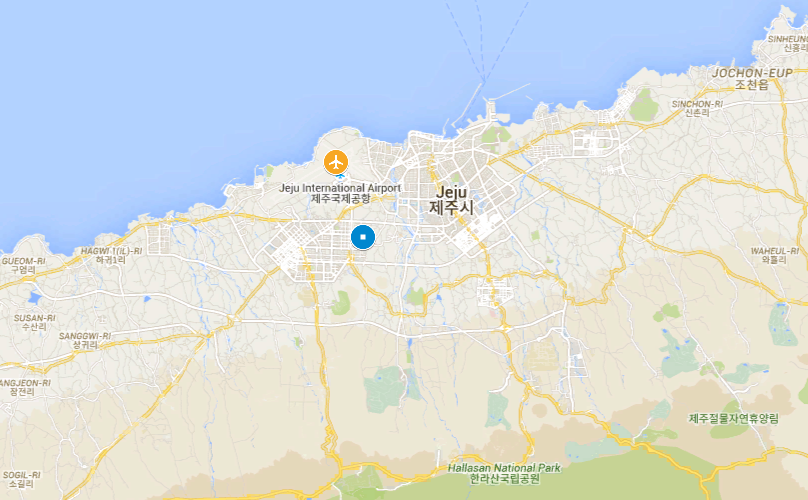


(b) Jeju, Korea


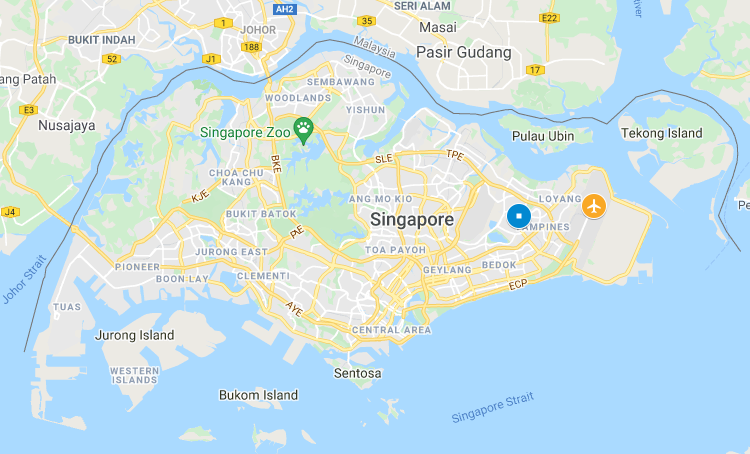


(c) Singapore

**Figure S2.** Overview of the three different regions—Incheon and Jeju in Korea and Singapore—included in the study (Map data ©2021 Google My Maps). Yellow circles indicate airports^*^ (Incheon, Jeju, and Changi International Airport, respectively) and blue circles indicate the nearest air-quality monitoring station to the airport in each case; *Unseo*, *Yeon-dong*, and *East*, respectively. The distance between the airport and air-quality monitoring station in Incheon, Jeju, and Singapore is 5, 3, and 5 km, respectively.

^*^Airport weather observations typically date back to when the airport was built; this abundance of data is beneficial for optimizing training data sets. Airport observations typically do not include air quality data; however, it is relatively easy to locate air quality monitoring stations near airports, since PM_2.5_ monitoring around urban areas is well-established in most countries since decades.

**Table S1.** Summary of meteorological parameters and PM_2.5_ concentration in Incheon and Jeju in Korea, and Singapore; the data range between January 2015 and December 2018 (i.e., 48 months) in the cases of Incheon and Jeju, and between April 2020 and September 2022 (i.e., 30 months) in the case of Singapore. All airport observations are complete; however, the PM_2.5_ data set consists of 33,364 observations from Incheon with 1,700 observations missing (i.e., missing rate 5%), 32,249 observations from Jeju with 2,815 observations missing (i.e., missing rate 8%), and 13,212 observations from Singapore with 8700 observations missing (i.e., missing rate 40%).

|  |  | $VIS$ (km) | ${PM}_{2.5}$  (μg m^-3^) | $Z_{{PM}_{2.5}}$ ^a^ | $TMP$ (℃) | $Z_{TMP}$ | $RH$ (%) | $Z_{RH}$ | $WS$ (kt)^b^ | $Z_{WS}$ |
| --- | --- | --- | --- | --- | --- | --- | --- | --- | --- | --- |
| (a)  Incheon | Min. | 0.1 | 0.0 | -1.35 | -16.0 | -2.68 | 8.3 | -2.98 | 0.0 | -1.72 |
|  | 1st Qu. | 7.0 | 10.0 | -0.70 | 3.3 | -0.84 | 48.8 | -0.75 | 4.0 | -0.79 |
|  | Median | 10.0 | 18.0 | -0.18 | 12.8 | 0.06 | 63.6 | 0.07 | 7.0 | -0.10 |
|  | Mean | 8.4 | 20.8 | 0.00 | 12.2 | 0.00 | 62.4 | 0.00 | 7.4 | 0.00 |
|  | 3rd Qu. | 10.0 | 29.0 | 0.53 | 21.2 | 0.86 | 77.6 | 0.84 | 10.0 | 0.60 |
|  | Max. | 10.0 | 159.0 | 8.94 | 36.6 | 2.32 | 97.8 | 1.95 | 33.0 | 5.93 |
| (b)  Jeju | Min. | 0.1 | 0.0 | -1.34 | -5.7 | -2.74 | 12.0 | -3.70 | 0.0 | -1.56 |
|  | 1st Qu. | 8.0 | 11.0 | -0.68 | 9.2 | -0.84 | 55.6 | -0.75 | 4.0 | -0.77 |
|  | Median | 10.0 | 18.0 | -0.26 | 16.1 | 0.04 | 66.9 | 0.02 | 6.0 | -0.37 |
|  | Mean | 8.8 | 22.3 | 0.00 | 15.8 | 0.00 | 66.6 | 0.00 | 7.9 | 0.00 |
|  | 3rd Qu. | 10.0 | 29.0 | 0.40 | 22.0 | 0.80 | 78.0 | 0.77 | 11.0 | 0.62 |
|  | Max. | 10.0 | 175.0 | 9.19 | 35.8 | 2.56 | 100.0 | 2.26 | 45.0 | 7.34 |
| (c)  Singapore | Min. | 1.0 | 1.0 | -1.60 | 22.0 | -2.80 | 43.0 | -3.81 | 0.0 | -1.64 |
|  | 1st Qu. | 10.0 | 7.0 | -0.70 | 26.0 | -0.89 | 74.0 | -0.70 | 3.0 | -0.73 |
|  | Median | 10.0 | 10.0 | -0.25 | 28.0 | 0.07 | 84.0 | 0.31 | 5.0 | -0.12 |
|  | Mean | 9.6 | 11.7 | 0.00 | 27.9 | 0.00 | 80.9 | 0.00 | 5.4 | 0.00 |
|  | 3rd Qu. | 10.0 | 15.0 | 0.50 | 29.0 | 0.54 | 89.0 | 0.81 | 2.0 | 0.49 |
|  | Max. | 10.0 | 68.0 | 8.48 | 34.0 | 2.93 | 100.0 | 1.91 | 22.0 | 5.05 |

^a^ *Z* is the standardized variable of the sub-titled.
^b^ 1 kt = 0.5144 m s^-1^; knots (kt), the unit for wind speed, is used in this study, as reported at the airport.


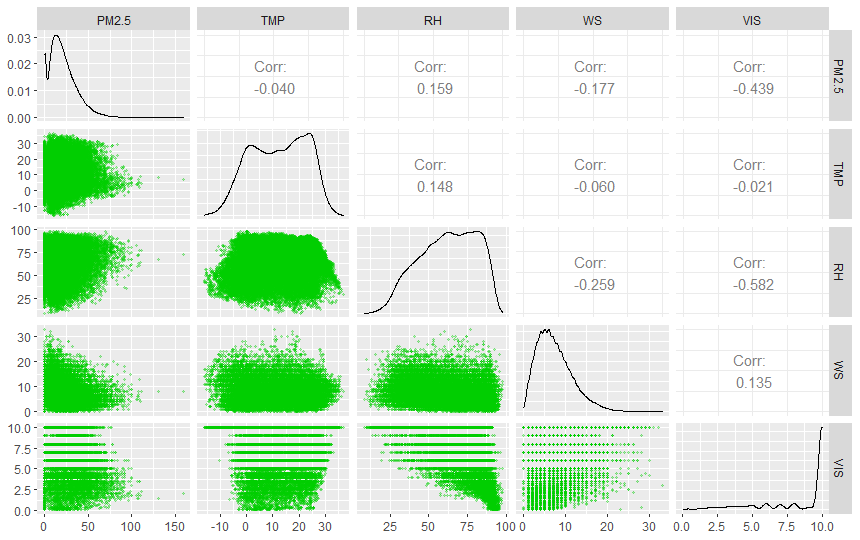


(a) Incheon, Korea


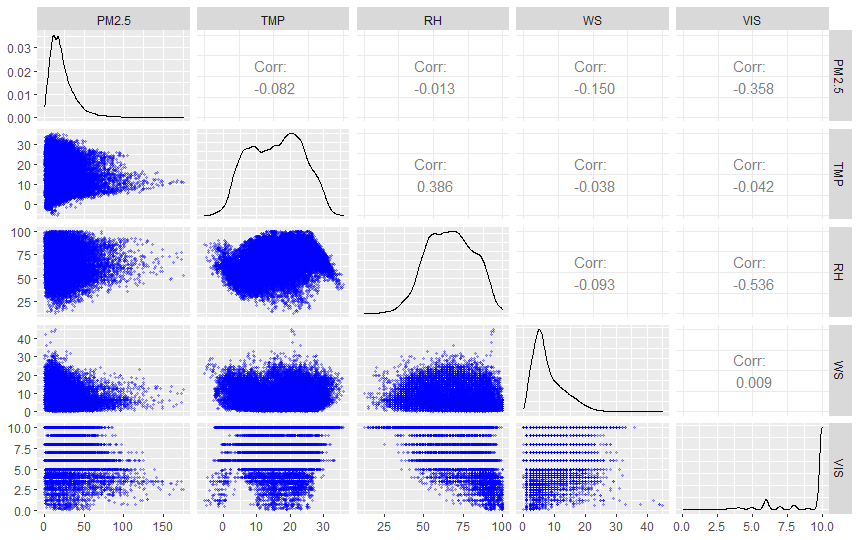


(b) Jeju, Korea


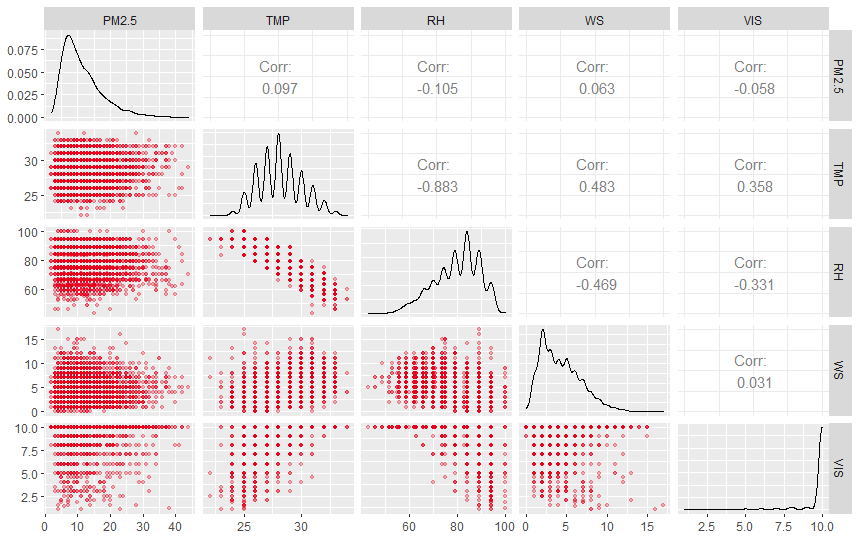


(c) Singapore

**Figure S3.** Correlation matrix between weather variables and visibility in (a) Incheon and (b) Jeju in Korea and in (c) Singapore: Relative humidity (RH) and PM_2.5_ concentration are the most negatively correlated with visibility in Incheon (-0.58 and -0.44, respectively) and Jeju (-0.54 and -0.36, respectively), and air temperature (TMP) and RH are the most correlated with visibility in Singapore (0.36 and -0.33, respectively).

**Table S2.** Variables applied to model design and degrees of freedom (df) of each model. Plus signs indicate when the variable is incorporated in the model and minus signs when it is not. In the present study, Model 2 was adopted.

| **Model**  **No.** | TMP | RH | WS | WX^*^ | PM_2.5_ | PM_2.5_ interactions | df |
| --- | --- | --- | --- | --- | --- | --- | --- |
| 0 | + | + | + | + | – | – | 12 |
| 1 | + | + | + | + | + | – | 13 |
| 2 | + | + | + | + | + | + | 16 |

^*^ WX (*present weather*) has eight levels: None, FG (fog), BR (mist), HZ (haze), DU (dust), DZ (drizzle), RA (rain), and SN (snow), which has the value of 0 or 1. *Present weather* is defined as the weather existing at the time of observation, or under certain conditions, during the hour preceding the time of observation (WMO-No. 306, 2017).

**Table S3.** Akaike information criterion (AIC) of the models based on the training data set and root mean squared error (RMSE) between modeled and observed visibility (km) based on the testing data set in (a) Incheon and (b) Jeju in Korea, and in (c) Singapore: The training data set ranges between January 2015 and December 2018 (i.e., 4 years) in Incheon and Jeju, and between April and November 2020 (i.e., 8 months) in Singapore. The testing data set ranges between January and December 2019 in Incheon and Jeju, and between December 2020 and January 2021 in Singapore.

| **Model**  **No.** | AIC (Training data set) | | | RMSE (Testing data set) | | |
| --- | --- | --- | --- | --- | --- | --- |
|  | (a) Incheon | (b) Jeju | (c) Singapore | (a) Incheon | (b) Jeju | (c) Singapore |
| 0 | 49619 | 39553 | 14329 | 2.12 | 2.09 | 2.27 |
| 1 | 47483 | 37879 | **14282** | 1.72 | 1.81 | **2.26** |
| 2 | **47179** | **37406** | **14282** | **1.69** | **1.78** | **2.26** |

**Table S4.** Modeled visibility according to model No. 2 of Table S2 and S3.

1. Incheon model (January 2015–December 2018)

|  | Estimate | Std. Error | z-value | p-value^a^ |  |
| --- | --- | --- | --- | --- | --- |
| (Intercept):1 | 7.505 | 0.047 | 159.675 | < 2e-16 | *** |
| (Intercept):2 | 0.607 | 0.007 | 89.727 | < 2e-16 | *** |
| $Z_{TMP}$ | 0.134 | 0.024 | 5.630 | 0.000 | *** |
| $Z_{RH}$ | -2.310 | 0.039 | -59.344 | < 2e-16 | *** |
| $Z_{WS}$ | -0.034 | 0.019 | -1.732 | 0.083 | . |
| $DU$ | -0.189 | 0.131 | -1.442 | 0.149 |  |
| $BR$ | 0.496 | 0.060 | 8.338 | < 2e-16 | *** |
| $FG$ | -2.617 | 0.107 | -24.528 | < 2e-16 | *** |
| $DZ$ | -1.453 | 0.164 | -8.886 | < 2e-16 | *** |
| $RA$ | 0.490 | 0.073 | 6.753 | 0.000 | *** |
| $SN$ | -0.036 | 0.137 | -0.262 | 0.793 |  |
| $Z_{{PM}_{2.5}.}$ | -1.004 | 0.023 | -44.585 | < 2e-16 | *** |
| $Z_{TMP}:Z_{{PM}_{2.5}}$ | -0.173 | 0.018 | -9.820 | < 2e-16 | *** |
| $Z_{RH}:Z_{{PM}_{2.5}}$ | 0.281 | 0.021 | 13.171 | < 2e-16 | *** |
| $Z_{WS}:Z_{{PM}_{2.5}}$ | 0.020 | 0.015 | 1.334 | 0.182 |  |

1. Jeju model (January 2015–December 2018)

|  | Estimate | Std. Error | z-value | p-value^a^ |  |
| --- | --- | --- | --- | --- | --- |
| (Intercept):1 | 8.199 | 0.067 | 122.430 | < 2e-16 | *** |
| (Intercept):2 | 0.582 | 0.008 | 77.058 | < 2e-16 | *** |
| $Z_{TMP}$ | 0.319 | 0.027 | 11.778 | < 2e-16 | *** |
| $Z_{RH}$ | -2.262 | 0.032 | -70.369 | < 2e-16 | *** |
| $Z_{WS}$ | -0.243 | 0.019 | -12.796 | < 2e-16 | *** |
| $DU$ | 0.636 | 0.156 | 4.075 | 0.000 | *** |
| $BR$ | 0.355 | 0.074 | 4.773 | 0.000 | *** |
| $FG$ | -3.078 | 0.160 | -19.204 | < 2e-16 | *** |
| $DZ$ | -1.040 | 0.209 | -4.968 | 0.000 | *** |
| $RA$ | 0.900 | 0.081 | 11.126 | < 2e-16 | *** |
| $SN$ | -0.553 | 0.132 | -4.200 | 0.000 | *** |
| $Z_{{PM}_{2.5}.}$ | -0.958 | 0.021 | -46.193 | < 2e-16 | *** |
| $Z_{TMP}:Z_{{PM}_{2.5}}$ | -0.186 | 0.020 | -9.158 | < 2e-16 | *** |
| $Z_{RH}:Z_{{PM}_{2.5}}$ | 0.374 | 0.018 | 20.329 | < 2e-16 | *** |
| $Z_{WS}:Z_{{PM}_{2.5}}$ | 0.171 | 0.014 | 11.904 | < 2e-16 | *** |

1. Singapore model (April 2020–September 2022)

|  | Estimate | Std. Error | z-value | p-value^a^ |  |
| --- | --- | --- | --- | --- | --- |
| (Intercept):1 | 9.839 | 0.009 | 1045.605 | < 2e-16 | *** |
| (Intercept):2 | -0.075 | 0.008 | -9.313 | < 2e-16 | *** |
| $Z_{TMP}$ | 0.112 | 0.018 | 6.169 | 0.000 | *** |
| $Z_{RH}$ | -0.130 | 0.019 | -6.777 | 0.000 | *** |
| $Z_{WS}$ | -0.065 | 0.010 | -6.184 | 0.000 | *** |
| $HZ$ | -5.134 | 0.144 | -35.688 | < 2e-16 | *** |
| $BR$ | -5.207 | 0.160 | -32.522 | < 2e-16 | *** |
| $RA$ | -1.890 | 0.029 | -66.205 | < 2e-16 | *** |
| $Z_{{PM}_{2.5}.}$ | -0.079 | 0.009 | -8.964 | < 2e-16 | *** |
| $Z_{TMP}:Z_{{PM}_{2.5}}$ | -0.029 | 0.017 | -1.719 | 0.086 | . |
| $Z_{RH}:Z_{{PM}_{2.5}}$ | -0.028 | 0.019 | -1.495 | 0.135 |  |
| $Z_{WS}:Z_{{PM}_{2.5}}$ | 0.047 | 0.010 | 4.621 | 0.000 | *** |

Part II. Application of the visibility-based calibration model

The LCM field testing was conducted over seven months, from March 25 to October 26, 2019, in Jeju, and over 22 months, from December 1, 2020 to September 30, 2022, in Singapore with the *‘W-Station’* of *Observer Foundation*. LCM specifications are provided in Figure S4 and Table S5; locations are provided in Figure S5. The *‘W-Station’* is equipped with the Sensirion SP30 PM_2.5_ sensor, temperature and humidity sensors, and a Wi-Fi transmitter, which transmits the data every 300 s. The fan inside the *‘W-Station’* generates airflow from below, the PM_2.5_ sensor measures the PM_2.5_ concentration by light scattering, and finally the air flows out in all directions. The weather data—such as TMP and RH—and PM_2.5_ concentration are transmitted to the server through Wi-Fi in real-time. In Singapore, we installed two LCMs side by side for estimating instrument precision (Figure S6).


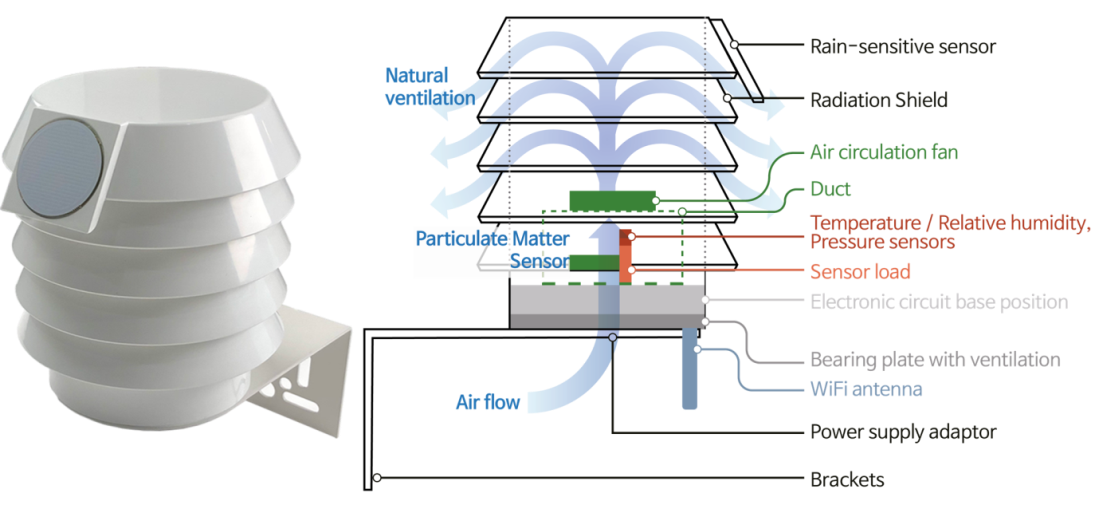


Figure S4. Exterior and interior of the low-cost monitor *‘W-station*’ (by *Observer Foundation*). The weather data and PM_2.5_ concentration are transmitted to the server through Wi-Fi in real-time. Measurements are available every minute, while the measuring interval can be adjusted manually. For our purposes, the data were recorded at 5-minute intervals and averaged hourly for comparing with the data from the reference monitoring systems.

**Table S5.** Specifications of the *‘W-station’* (by *Observer Foundation*).

| Parameter | Condition | Value | |
| --- | --- | --- | --- |
| Particulate matter sensor | Sensor Type | Laser-based light scattering particle sensing | |
|  | Mass range | 0–1,000 μg m^-3^ | |
|  | Accuracy (<100) | 0–100 μg m^-3^ | ±10 μg m^-3^ |
|  | Accuracy (>100) | 100–1,000 μg m^-3^ | ±10% |
|  | Size range (PM_2.5_) | 0.3 to 2.5 μm | |
|  | Ventilation | Fan aspirated | |
| Temperature | Type | PT100 | |
|  | Operating Range | -40 to +60℃ | |
|  | Accuracy | ±0.3 ℃ | |
| Humidity | Type | Capacitive | |
|  | Operating Range | 0–100% RH | |
|  | Accuracy | ±3.0% RH @ 25 ℃ (10–90% RH) | |


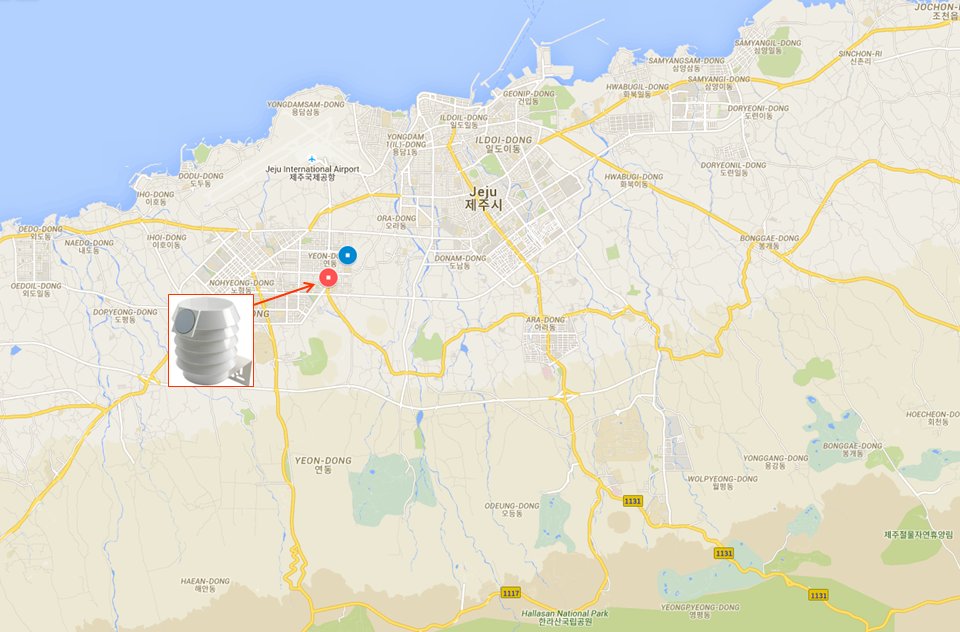


(a) Jeju, Korea


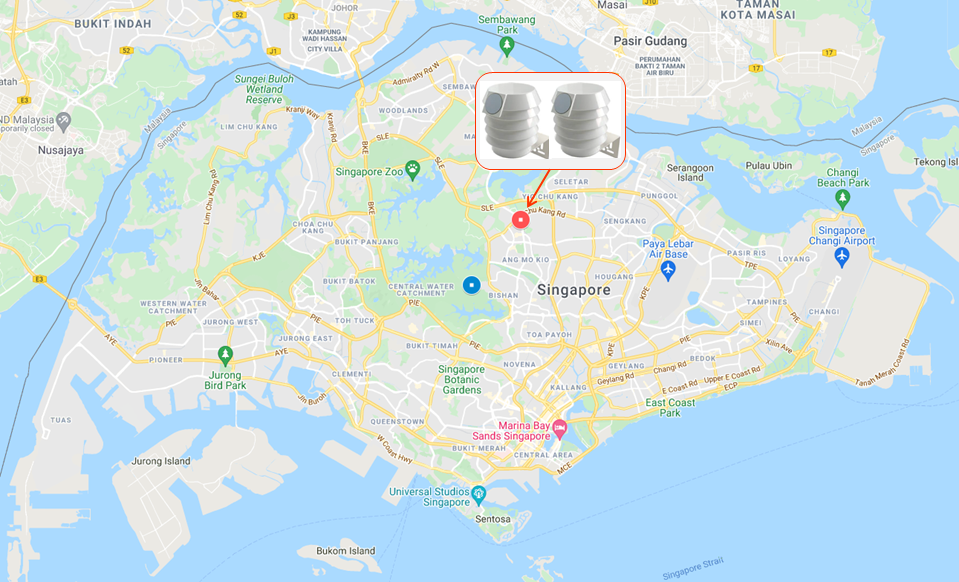


(b) Singapore

**Figure S5.** LCM field testing locations (red circle marks) and the nearest reference station, *Yeon-dong* and *Central* (blue circle marks) in (a) Jeju and (b) Singapore (Map data ©2021 Google My Maps). The distances between the low-cost sensor and the reference station are 0.6 km and 4.2 km in Jeju and Singapore, respectively. PM_2.5_ measurements were conducted over seven months (March 25 to October 26, 2019) in Jeju and over 22 months (December 1, 2020 to September 30, 2022) in Singapore.


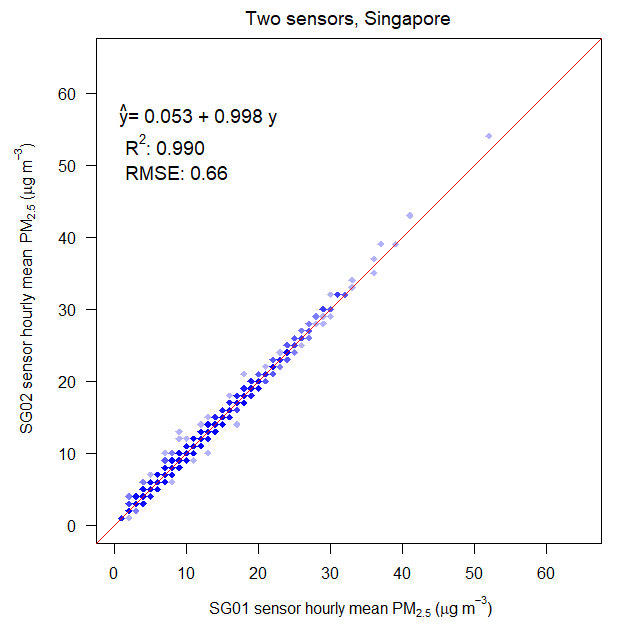

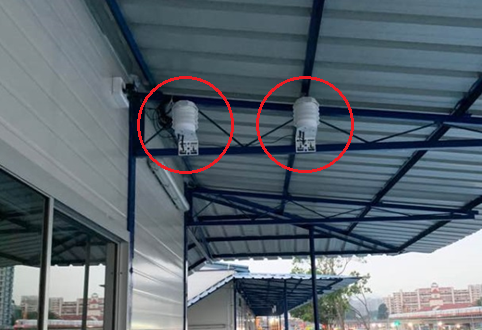


**Figure S6.** Comparison of two LCMs that were installed 50 cm apart in Singapore: Results indicate high precision between them (R^2^ = 0.99; RMSE = 0.66 μg m^-3^).

**Table S6.** Selected weather observations and PM_2.5_ concentration (μg m^-3^) before and after calibration in Jeju, Korea on March 29, 2019.

| DATE_TIME | WD  (0–360°) | WS  (knot) | TMP  (℃) | RH  (%) | PM_2.5_ before calibration | PM_2.5_ after calibration | PM_2.5_ by reference station |
| --- | --- | --- | --- | --- | --- | --- | --- |
| 2019-03-29 0:00 | 360 | 2 | 14 | 73 | 86 | **72** | **60** |
| 2019-03-29 1:00 | 70 | 3 | 14 | 74 | 76 | **64** | **50** |
| 2019-03-29 2:00 | 220 | 4 | 13 | 75 | 75 | **62** | **50** |
| 2019-03-29 3:00 | 170 | 4 | 13 | 74 | 85 | **71** | **65** |
| 2019-03-29 4:00 | 150 | 2 | 11 | 74 | 75 | **64** | **62** |
| 2019-03-29 5:00 | 220 | 2 | 11 | 76 | 74 | **61** | **61** |
| 2019-03-29 6:00 | 260 | 3 | 11 | 77 | 71 | **57** | **58** |
| 2019-03-29 7:00 | 330 | 1 | 12 | 77 | 65 | **52** | **51** |
| 2019-03-29 8:00 | 280 | 2 | 13 | 73 | 41 | **37** | **39** |
| 2019-03-29 9:00 | 280 | 4 | 14 | 69 | 38 | **37** | **38** |
| 2019-03-29 10:00 | 310 | 6 | 17 | 59 | 33 | **36** | **37** |
| 2019-03-29 11:00 | 70 | 2 | 16 | 53 | 27 | **31** | **31** |
| 2019-03-29 12:00 | 10 | 4 | 19 | 46 | 25 | **31** | **28** |


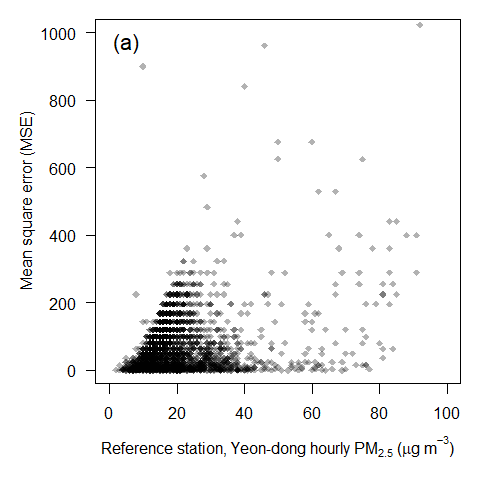

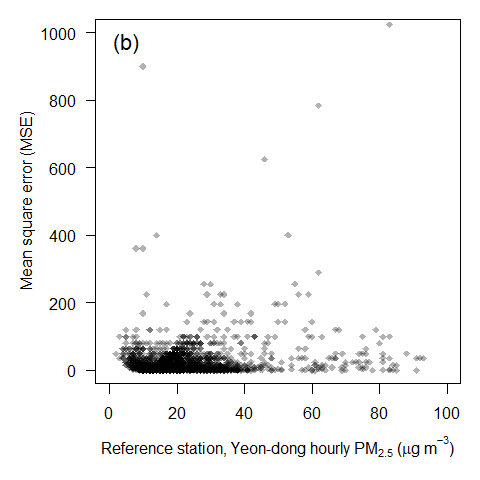


**Figure S7.** Mean-square error (MSE) between PM_2.5_ concentration measured by the LCM and measurements of the reference station, *Yeon-dong* in Jeju, Korea: (a) Raw PM_2.5_ data of the LCM and (b) post-processed data according to the calibration method.


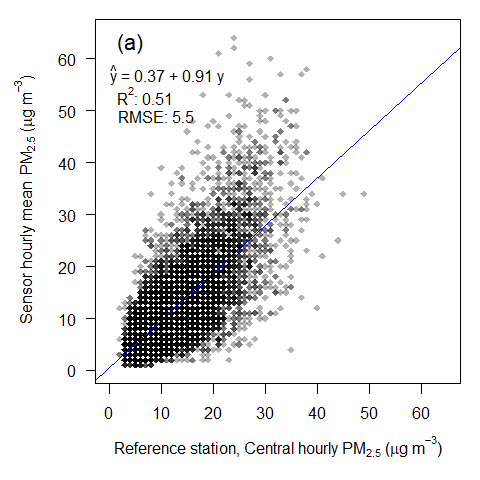

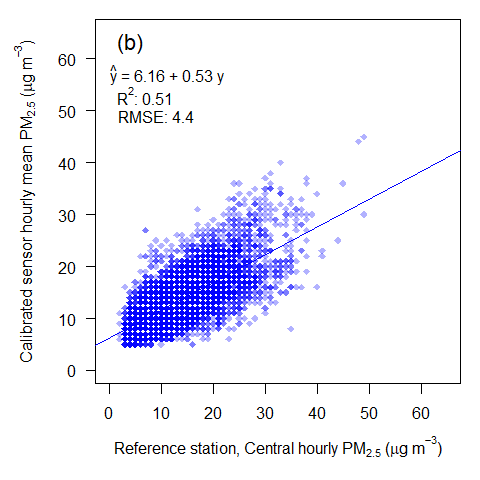


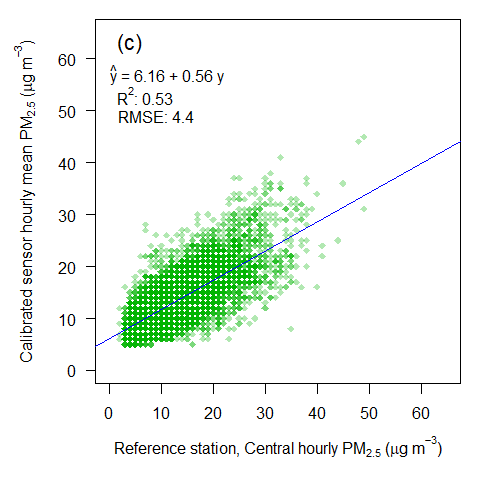

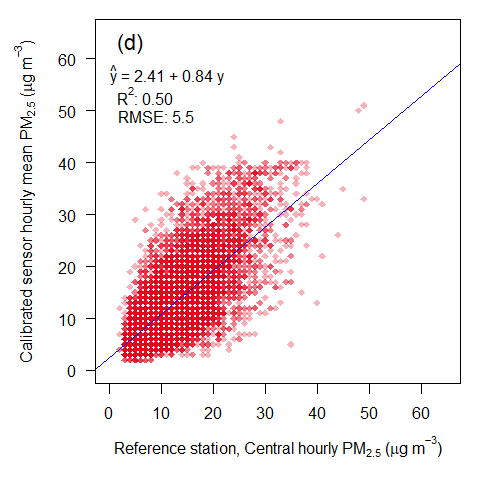


**Figure S8.** Scatterplots of hourly PM_2.5_ mass concentration of the LCM against the hourly measurements of the reference station in Singapore (December 1, 2020 to September 30, 2022): (a) Raw PM_2.5_ data of the LCM; post-processed data according to the calibration method of the (b) Jeju, (c) Incheon, and (d) Singapore model.
